# A20 enhances the radiosensitivity of hepatocellular carcinoma cells to ^60^Co-γ ionizing radiation

**DOI:** 10.18632/oncotarget.21860

**Published:** 2017-10-16

**Authors:** Rui Liu, Dongli Zhao, Xiaozhi Zhang, Suxia Han, Yunyi Yang, Jinlu Ma, Du Meng

**Affiliations:** ^1^ Department of Radio Oncology, The First Affiliated Hospital of Xi’an Jiaotong University School of Medicine, Xi’an 710061, The People's Republic of China

**Keywords:** hepatocellular carcinoma, A20, ^60^Co-γ ionizing radiation, radiotherapy, radiosensitization

## Abstract

The radioresistance of hepatocellular carcinoma (HCC) cells is a critical obstacle for effectively applying radiotherapy (RT) in HCC treatment. NF-κB, an important transcription factor, can influence critical cell fate decisions by promoting cell survival or anti-apoptosis in response to cell-stress, *e.g.* chemotherapies or ionizing radiation (IR). A20, also named as tumor necrosis factor α induced protein 3 (*TNFAIP3*), is a dominant negative regulator of NF-κB pathway and its functions in HCC are largely unknown. The present work aimed to reveal the role of A20 plays in affecting the radiosensitivity of HCC cells. Higher expression of A20 was detected in hepatic non-tumor cell line or clinical specimens compared with HCC cell lines or clinical specimens. A20 decreased the expression of proteins mediating cellular stress/injury response or epithelial-mesenchymal transition (EMT) process. Overexpression of A20 *via* adenovirus enhanced the effect of ^60^Co-γ ionizing radiation (IR) on HCC cells’ injury, *e.g.* G2/M arrest or DNA double strands break (DSB). Moreover, A20 also enhanced the *in vitro* or *in vivo* survival inhibiting of HCC cells induced by IR. These results reveal the roles of A20 in HCC radiosensitization and overexpression of A20 would be a novel strategy for HCC radiotherapy.

## INTRODUCTION

Liver diseases are a heavily medical burden in Asian-pacific region, especially in China [1]. Considerable proportion of patients infect with HBV or are from HBV related chronic hepatitis would finally progress into hepatocellular carcinoma (HCC), an end-stage liver diseases (ESLD), even with efficient anti-viral treatment [1, 2]. Although hepatic resection is a well-established therapeutic method, it is only suitable for low proportion (10-15%) of HCC patients [3, 4]. Most patients are suffering from advanced HCC at the initial diagnosis, and exhibit poor prognosis after chemotherapy, due to the multi-drug resistance (MDR) of HCC cells to chemotherapeutical agents [5, 6]. Recently, oral multi-target kinase inhibitors, such as sorafenib, were showed to attenuate HCC progress or metastasis; however, the five-year survival rate of HCC patients is still low [7–10]. The initial or acquired resistance to sorafenib has also drawn attention [11]. To date, radiotherapy is still a preferred treatment for advanced HCC and the novel radiotherapy strategies seem promising [12, 13]. However, tumor radioresistance to radiation agents such as ionizing radiation (IR) remains an obstacle in effective HCC treatment [14, 15]. Some local-ablation strategies, *e.g.* radiofrequency ablation (RFA), transcatheter arterial chemoembolization (TACE) or cryoablation, would induce body injury via percutaneous puncture and have the risks to induce the metastasis [16–18]. Therefore, it is valuable to develop novel strategies that can be effectively enhance radio-therapeutic efficacy for treating HCC.

In cancer cells, NF-κB is an important transcription factor that are activated by many intro-cellular or extra-cellular signals pathways, *e.g.* Notch-1. When cells were in response to cell-stress or injury, Notch-1 can be activated by cleaving and in turn activates NF-κB [19, 20]. Activated NF-κB promotes anti-apoptosis or pro-survival by mediates some targeted genes’ expression, such as Bcl-2, Cyclin D1, cIAPs (cellular inhibitor of apoptosis, cIAPs) or Survivin [21, 22]. Some investigations have provided the clues that, inhibition of Notch-1/NF-κB activation may lead to anticancer effects [23, 24]. Kang et al., 2013 elucidated that blocked Notch-1/NF-κB pathway activation by novel chemical compounds may enhance the sensitivity of cancer cells to IR [23]. Therefore, NF-κB would be a useful therapeutic target and down-regulation of NF-κB's activation would be a useful strategy to overcome radioresistance of HCC cells and enhance the efficacy of radiotherapy in HCC treatment.

The ubiquitin regulator A20 (also named as tumor necrosis factor, alpha-induced protein 3, encoded by *TNFAIP3*) is a master suppressor of NF-κB [25–27]. Research works of A20 mainly focused on its role in immune regulation [28–32]: A20 can protect human bodies from some severe/aggressive immune reactions/process, *e.g.* allergic disorders. In this study, we showed that lower expression of A20 was identified in HCC cells or clinical specimens, compared with non-tumor hepatic cell line L-02 or non-tumor specimens. Overexpression of A20 in HCC cells *via* adenoviral vector enhanced injury induced by ^60^Co-γ ionizing radiation (IR). A20 also enhanced the *in vitro* or *in vivo* survival inhibiting of HCC cells induced by IR. Our study indicates that A20 could be a novel therapeutic strategy to increase radiotherapy efficiency in HCC treatment.

## RESULTS

### A20 would be involved in HCC regulation

**Figure 1:**
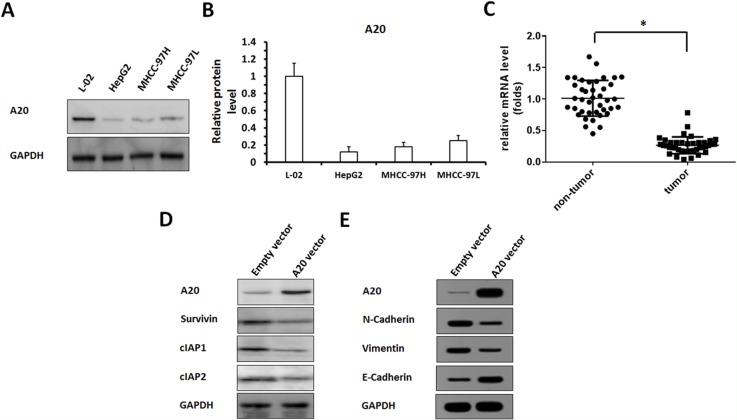
The expression of A20, and other proteins in HCC cells or clinical specimens **(A, B)** Protein samples extracted from cell lines were analyzed by western blot, and GAPDH was chosen as a loading control. Results was shown as protein banding patterns. (C) RNA samples extracted from HCC (n=40) and non-tumor (n=40) clinical specimens were analyzed by qPCR, and β-Actin was chosen as a loading control. **(D, E)** HepG2 cells infected with empty vector or A20 vector were harvested for western blot examination. (D) The expression of A20, Survivin, cIAP-1 and cIAP-2 were detected by antibodies. (E) The expression of A20, E-Cadherin, N-cadherin or Vimentin were detected by antibodies. GAPDH was used as a loading control. ^*^P<0.05.

Endogenous protein levels of A20 in HCC cells and non-tumor cells were showed in Figure 1A, 1B, lower level of A20 expression was detected in three HCC cell lines, HepG2, MHCC-97H and MHCC-97L, comparing to L-02 cells, a non-tumor liver cell line. Among these HCC cell lines, HepG2 cells expressed A20 at the lowest level. L-02 cells expressed A20 at the highest level. Therefore, we chose HepG2 cells to overexpress A20, and L-02 cells to knockdown A20 expression.

Next, the expression of A20 in HCC clinical specimens were detected. The mRNA level of A20 in HCC and adjacent non-tumor specimens (Table 1) was detected by qPCR. As shown in Figure 1C, a lower level of A20 was detected in HCC compared with adjacent non-tumor tissues.

**Table 1:** Baseline characteristics of patients in this stufy

Clinical features	Values
Age (yr)	51.4±7.50
Gender (number)	
Male	33
Female	7
Aetiology	
Hbs-Ag positive	35
HCV-Ab positive	5
Child-Pugh score	
Class A	38
Class B	2
Tumour size	
<3cm	30
3-5cm	10
Tumour number	
Single	21
2-3	19
BCLC staging	
Stage A	35
Stage B	5
Tumour differentiation	
well	13
moderate	19
poorly	8
AFP	501±690

To study the potential mechanism of A20 in HCC, we constructed the Adenovirus vectors to overexpress and knockdown A20. The results showed that overexpression of A20 reduced the level of pro-survival/anti-apoptosis regulators, Survivin, cIAP-1 and cIAP-2, in HepG2 cells (Figure 1D, Supplementary Figure 1). Moreover, the effect of A20 on HepG2 cells’ EMT process was detected. As shown in Figure 1E, Supplementary Figure 2, overexpression of A20 reduced the protein level of N-Cadherin or Vimentin, two mesenchymal markers, and enhanced epithelial marker E-Cadherin's expression. Therefore, A20 would be involved in HCC regulation and modulate pro-survival/anti-apoptosis regulators’ expression or EMT process.

### A20 Enhances the effect of ^60^Co-γ IR on cells

**Figure 2:**
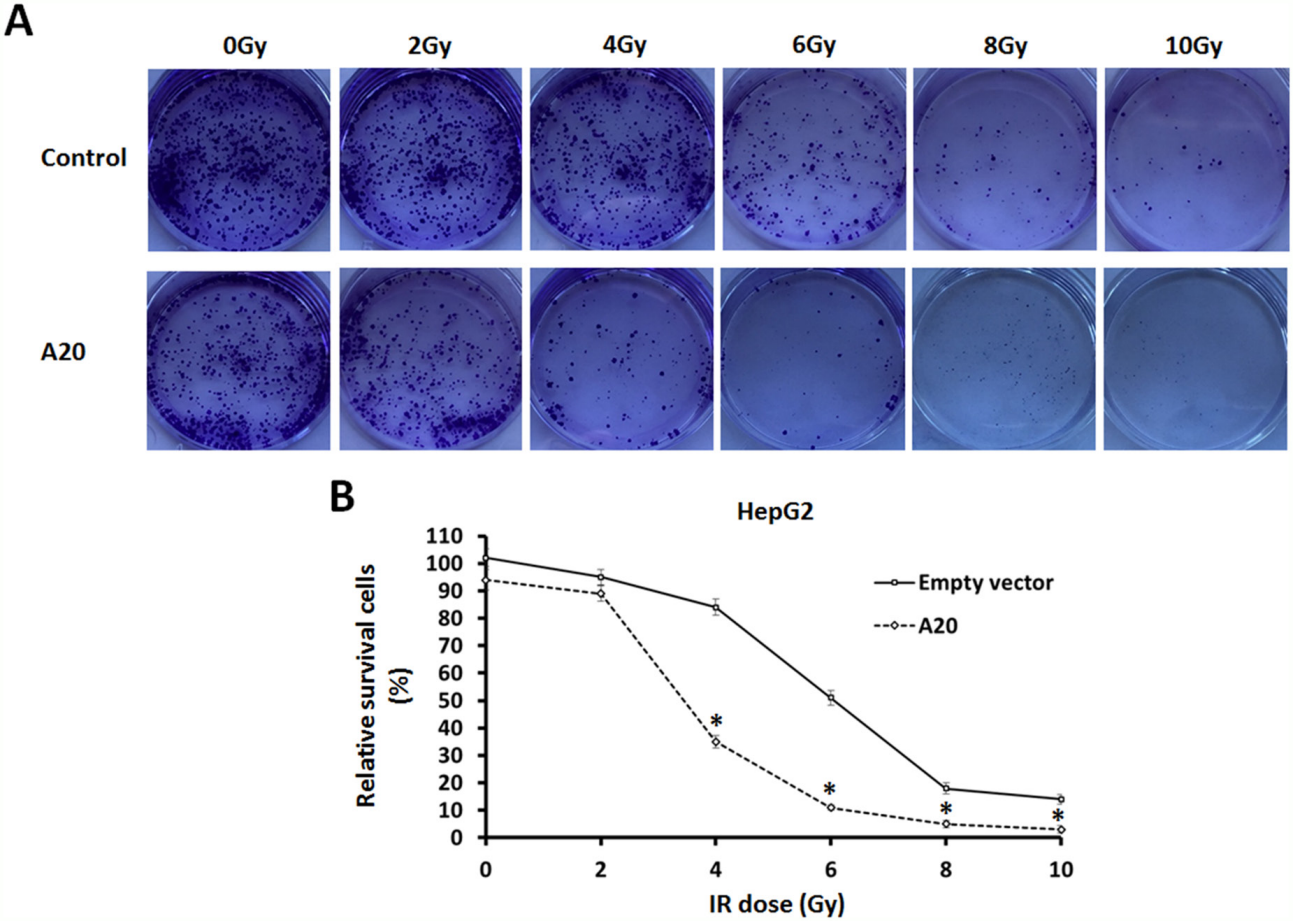
A20 enhances the sensitivity of HepG2 cells to ^60^Co-γ IR **(A)** HepG2 cells infected with empty vectors or A20 vectors were treated with 0, 2, 4, 6, 8 or 10Gy dose of 60Co-γ IR, were harvested and seed in 6-well plates (2 × 10^3^ cells per well). After cultured for 3–4 weeks, colonies were staining with crystal violet (0.5% in 20% ethanol). Then, colonies were harvested and measured by a multifunctional micro-plate reader at 546 nm. Results were shown as **(B)** typical photographs or relative colony number (mean ± SD). ^*^P<0.05.

Next, the effect of A20 on HCC cells’ radiosensitivity was determined. Our results showed that after 0, 2, 4, 6, 8 or 10Gy dose of ^60^Co-γ IR treatment, the colony formation of HepG2 cells was significantly attenuated as expected, and 6Gy dose of IR was a middle-effect dose. Interestingly, overexpression of A20 in HepG2 cells enhanced the anti-tumor activity of IR (Figure 2A, 2B).

To further confirm the specificity of A20 in radiosensitivity, we studied the effect of A20 knockdown in L-02, which is a non-tumor hepatic liver cell line and expresses high level of A20. As shown in Supplementary Figure 3, Knockdown of A20 attenuated the L-02 cells injury induced by 6Gy dose of IR. These results suggest that A20 may enhances the radiosensitivity of tumor cells.

### A20 enhances IR Induced injury of HCC cells

**Figure 3:**
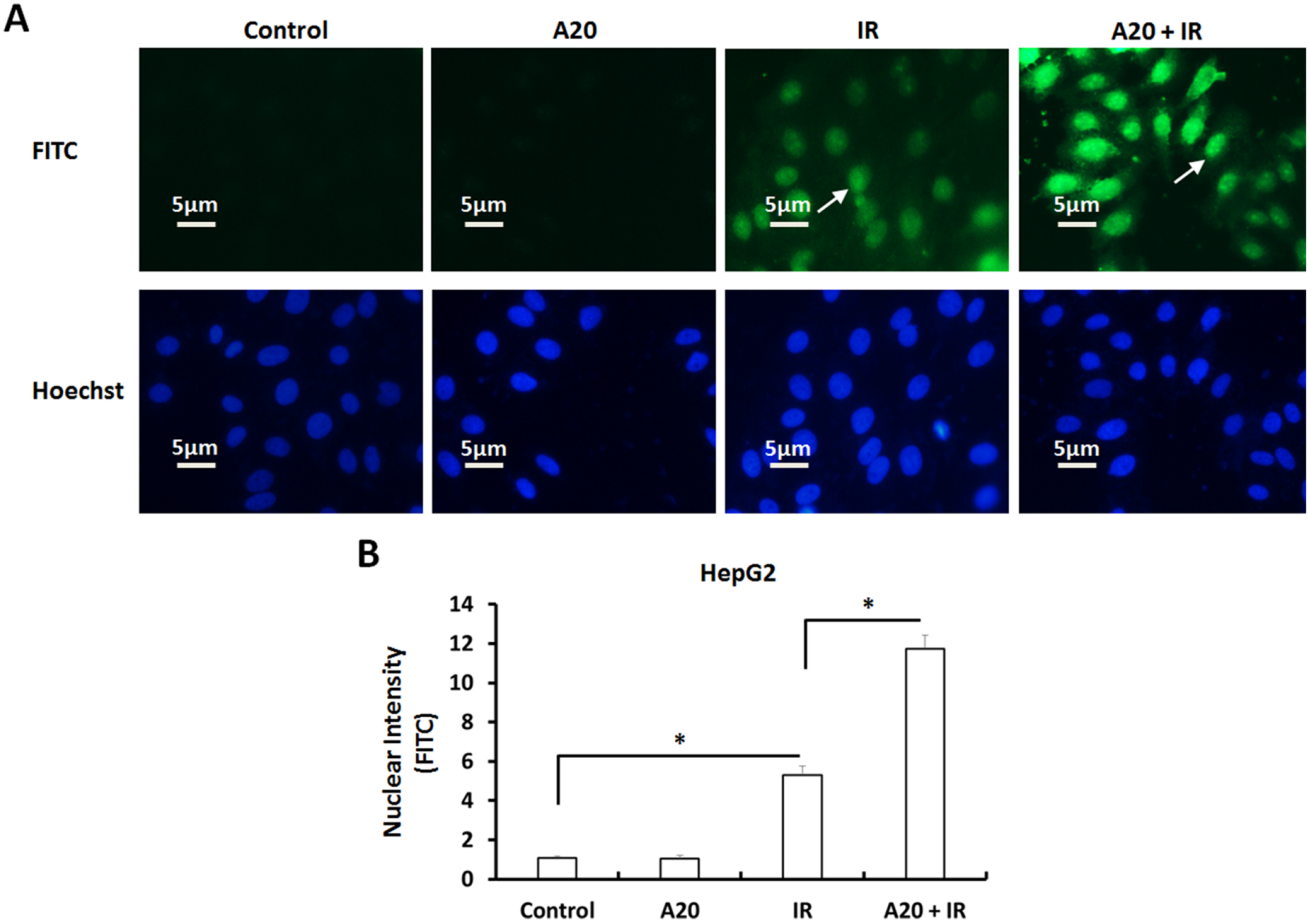
Overexpression of A20 prompts the DNA double strands break (DSB) induced by IR in HepG2 cells **(A, B)** HepG2 cell infected with empty vector or A20 vectors were treated without or with 6Gy dose of IR. After 30min, the TUNEL reactions were performed to examine the DSB (DNA double strand break). Results were shown as typical photograph (A) or nuclear intensity (B). The FITC-fluorescence indicated the DSB and Hoechst-fluorescence indicated the nuclear of HepG2 cells. ^*^P<0.05.

Further, we performed TUNEL assays to examine the DNA Double Strand Break (DSB) of HepG2 cells induced by IR. As shown in Figure 3, overexpression of A20 did not induce the DSB of HepG2 cells. Treatment of 6Gy dose IR induced the DSB of HepG2 cells (Figure 3A) and overexpression of A20 enhanced the effect of IR (Figure 3B).

**Figure 4:**
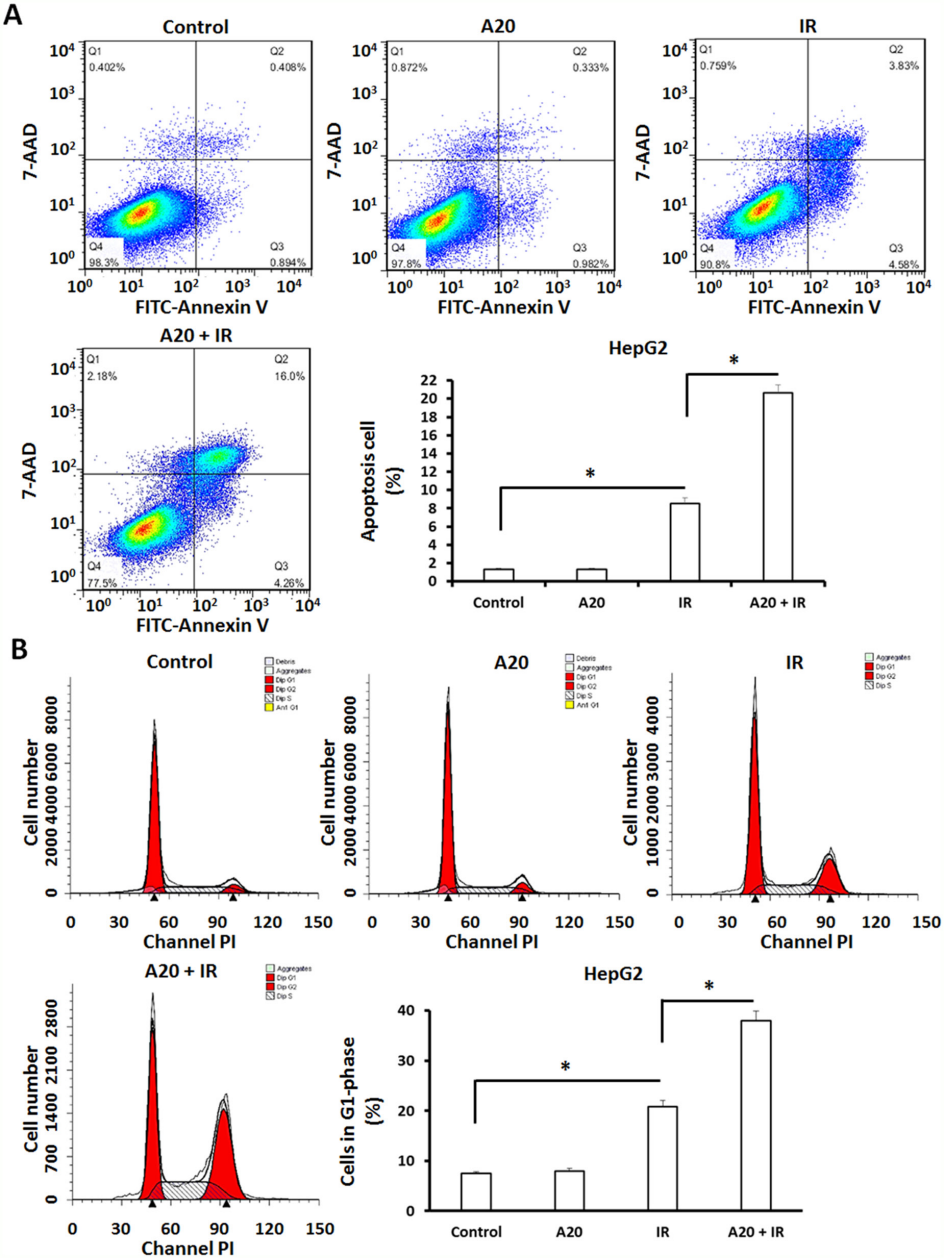
Overexpression of A20 prompts the apoptosis and G2/M arrest induced by IR in HepG2 cells HepG2 cells infected with empty vector or A20 vector were treated without or with 6Gy IR. **(A)** After 24h, cells were harvested for apoptosis analysis. Apoptotic HepG2 cells were shown as typical photograph or mean ± SD. **(B)** After 24h, cells were harvested for cell cycle analysis. Cell in G2/M phase were shown as typical photograph or mean ± SD. ^*^P<0.05.

To further examine the effect of A20, we performed flow cytometer assays to examine the apoptosis of HepG2 cells. As shown in Figure 4, overexpression of A20 did not induce the apoptosis or G2/M arrest of HepG2 cells. The proportion of apoptotic HepG2 cells was increased from 1.30% to 8.41% after 6Gy dose of IR treatment (Figure 4A). Consequently, overexpression of A20 up-regulated the effect from 8.41% to 20.26% (Figure 4A).

Given that cell cycle arrest is a specific feature of IR on tumor cells, the G2/M arrest of HepG2 cells received IR treatment was examined. As shown in Figure 4B, the proportion of HepG2 cells in G2/M was increased after 6Gy dose of IR treatment from 7.50% to 20.85%, overexpression of A20 unregulated this effect from 20.85% to 37.98%.

**Figure 5:**
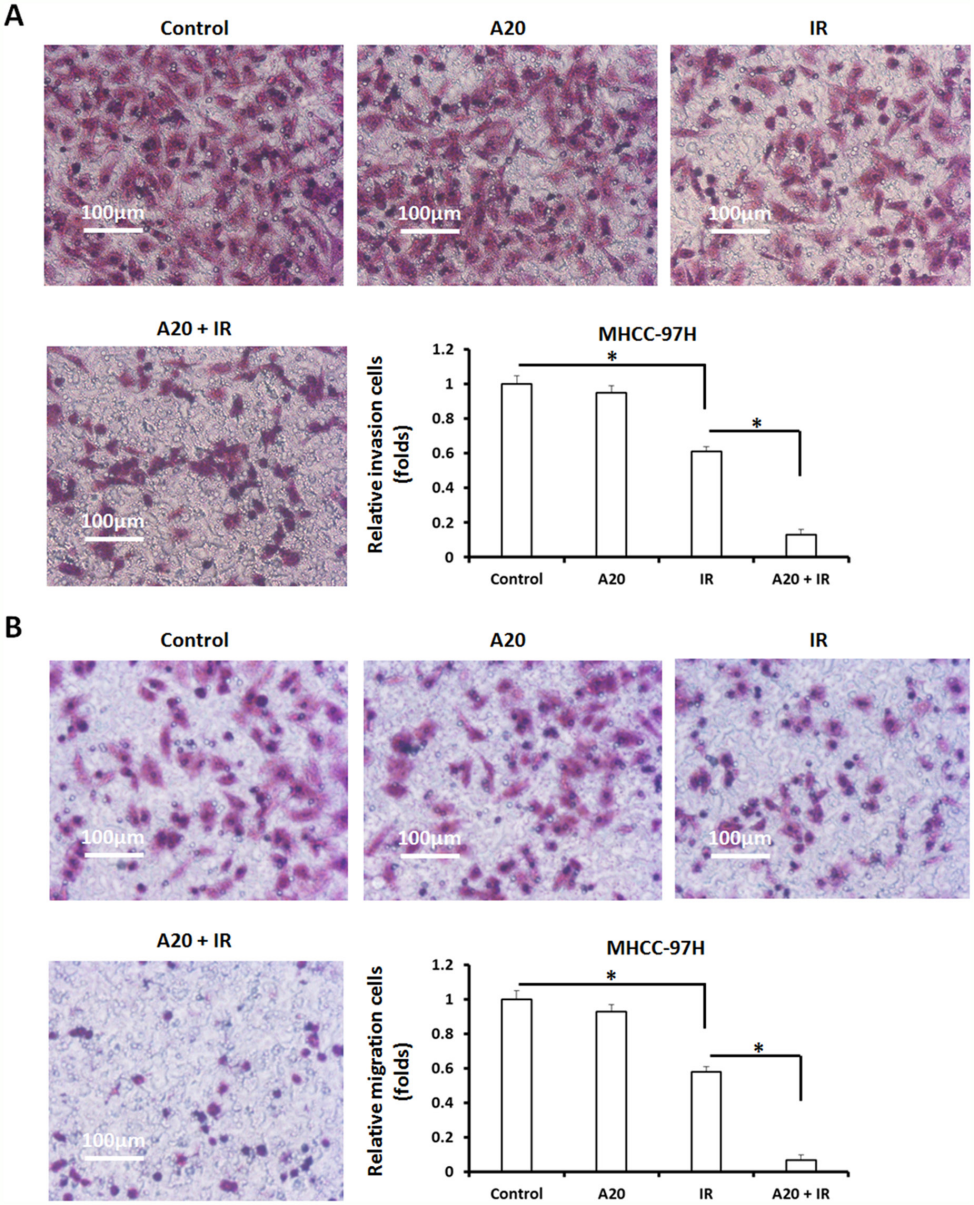
Overexpression of A20 expression in MHCC-097H cells promotes the inhibitory activation of IR on MHCC-97H cells’ *in vitro* invasion/migration **(A, B)** MHCC-97H cells infected with empty vectors or A20 vectors were treated with 6Gy dose of ^60^Co-γ IR, were harvested for transwell analysis. The *in vitro* invasion (A) or migration (B) of MHCC-97H was determined. Results were shown as typical photographs or relative colony number (mean ± SD). ^*^P<0.05.

Invasion and migration are main features of metastatic malignancies, and are associated with the progression or prognosis of human cancers. To test the effect of A20 in invasion/migration inhibiting induced by IR, a highly aggressive HCC cell line MHCC-97H was used. As shown in Supplementary Figure 4, 6Gy does IR treatment inhibited the colony formation of MHCC-97H cells. Overexpression of A20 enhanced the effect of IR on MHCC-97H's colony formation. Next, we performed transwell experiments. MHCC-97H cells infected with empty vector or A20, were treated with 6Gy dose of IR. As shown in Figure 5, IR attenuated *in vitro* invasion and migration of MHCC-97H cells and A20 enhanced this effect. Taken together, A20 enhances the effect of IR on HCC cells.

### A20 enhances *in vivo* antitumor efficiency of IR therapy

**Figure 6:**
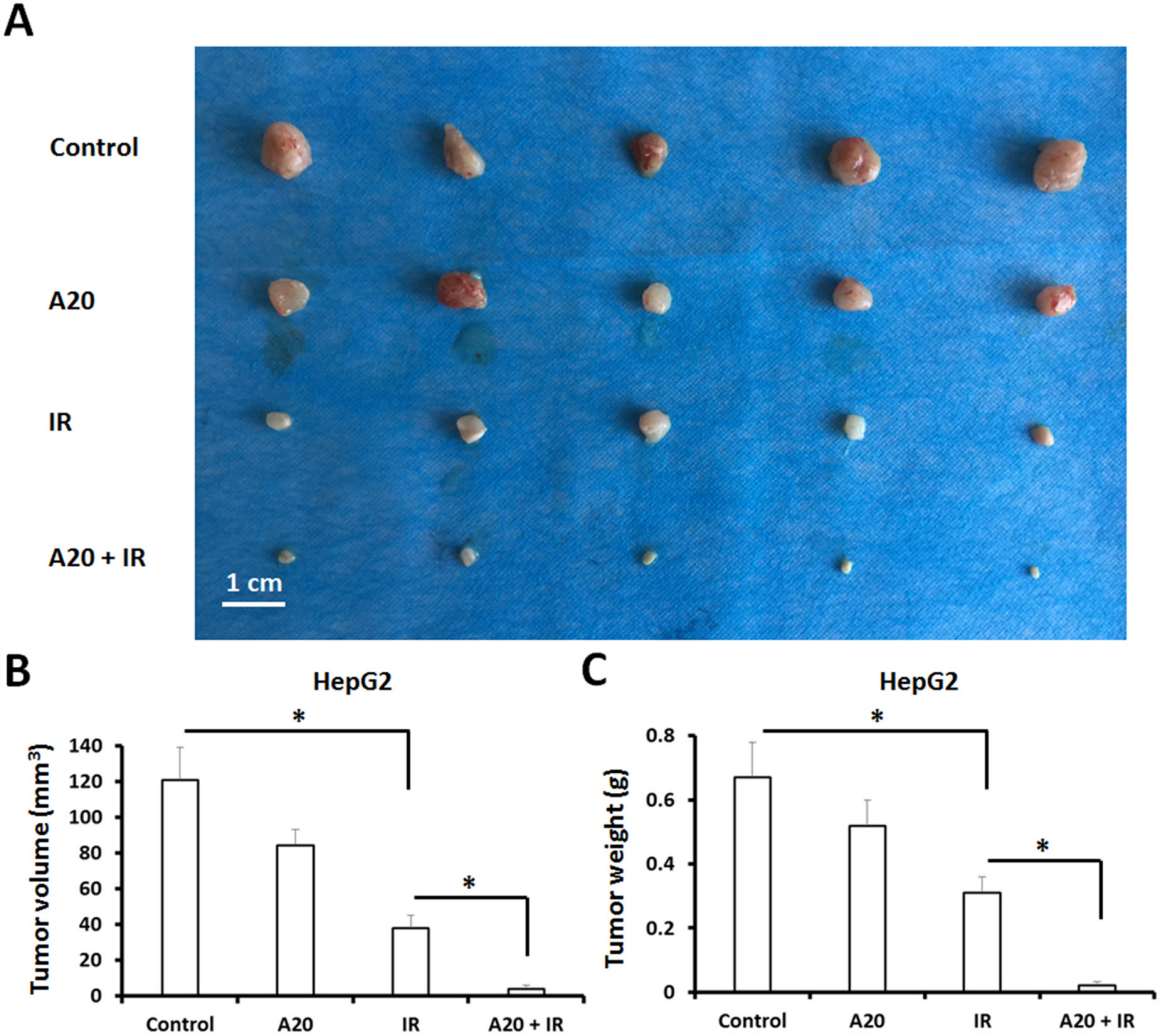
Overexpression of A20 enhances the sensitivity of HepG2 cells to IR via a nude mice subcutaneous tumor model **(A)** HepG2 cells infected with empty vector or A20 vector were treated without or with 6Gy dose of IR. After 24h, cells were injected into nude mice. After 4-6 weeks growth, the tumor volume and weight was determined. Results were shown as typical photograph (A) or mean ± SD **(B, C)**. ^*^P<0.05.

To identify the role of A20 in antitumor radiation therapy, HepG2 cells infected with empty vector or A20, were treated with 6Gy dose of IR and injected into nude mice. The results showed that 6Gy dose of IR inhibited the *in vivo* growth of HepG2 cells (Figure 6), and overexpression of A20 further enhanced this antitumor effect (Figure 6).

**Figure 7:**
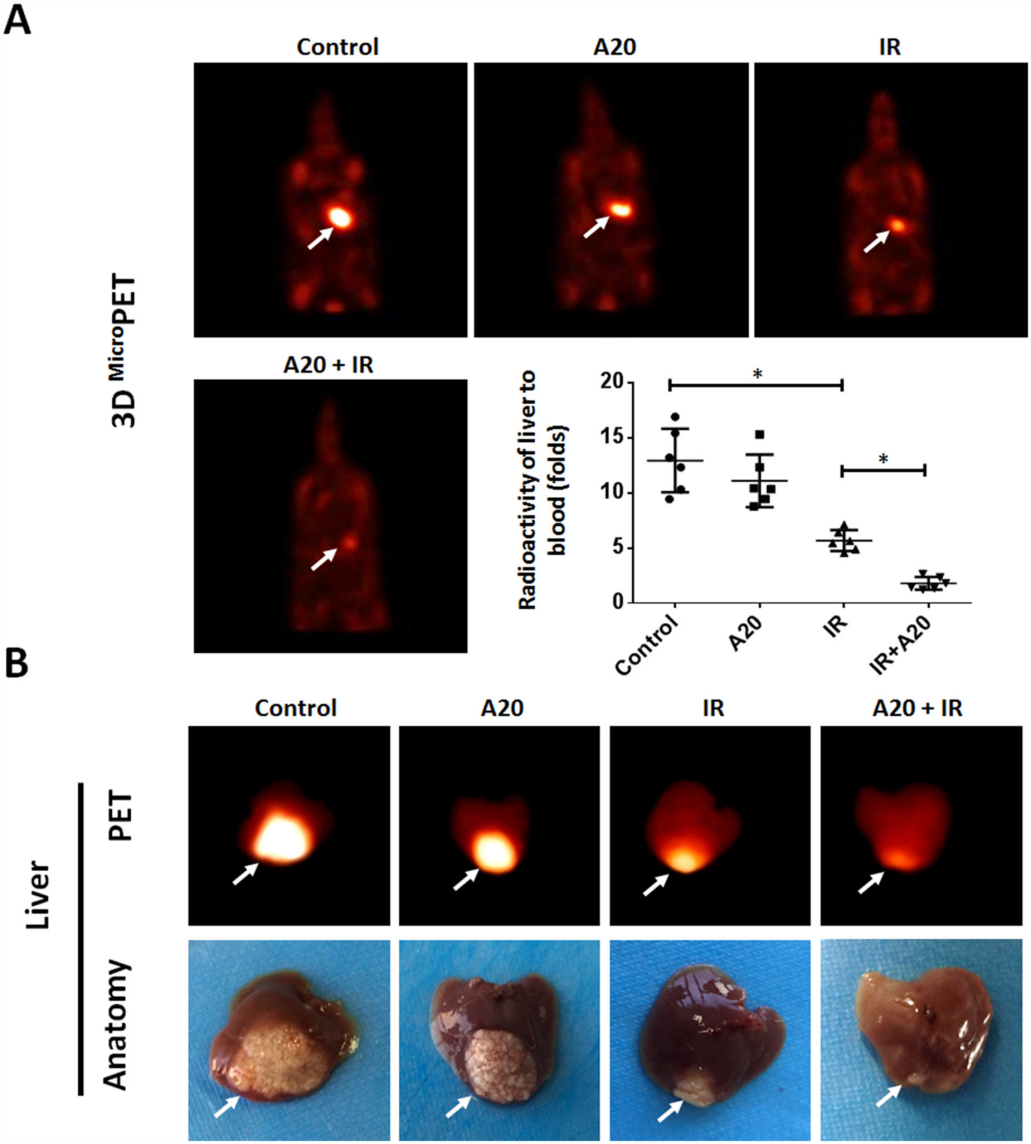
Overexpression of A20 enhances the sensitivity of HepG2 cells to IR via a nude mice intra-hepatic tumor model HepG2 cells infected with empty vector or A20 vector were treated without or with 6Gy dose of IR. After 24h, cells were injected into the right lobe of the liver. After 4-8 weeks growth, ^18^FDG-PET images were collected (n = 6) **(A)**. **(B)** Images and radioactivity of ablated livers were also showed. Arrows indicate tumor nodules. ^*^P<0.05.

Next, we tested the effect of A20 in an intra-hepatic / *in situ* liver tumor model. The effect of IR and A20 was measured by PET images and the intra-hepatic nodules region. As shown in Figure 7, PET imagines indicated the *in situ* tumor in liver. Treatment of IR clearly decreased the nodules formed by HepG2 in liver tissue (Figure 7A). Overexpression of A20 enhanced the effect inhibitory effect of IR on nodules region in liver tissue (Figure 7A). The liver-to-blood radioactive data, tumor foci in liver tissues and the PET imagine of liver confirmed the PET results from animals (Figure 7B). Taken together, our *in vitro* and *in vivo* data indicated that A20 enhances the radiosensitivity of HCC cells to IR treatment.

## DISCUSSION

Radiation therapy is a routine treating option for patients suffering from advanced HCC [33]. Compared to surgery, RFA, TACE or cryoablation, radiation therapy is a generally non-invasive therapy for advanced HCC [34]. However, the radioresistance of HCC remains to be a critical obstacle [35–39]. Clinical investigations have reported that the one-third, two-thirds or whole liver can only be safely irradiated with 90, 47 or 31Gy does of IR, respectively; however, these doses are only a partial volume of a HCC-control dose [35–39]. Although some advanced irradiation techniques, such as 3D-conformal RT (CRT) or image-guided RT (IGRT), can provide an enough dose HCC injury and reduce normal liver tissue damage, the efficiency of those approaches enough [35–39]. Therefore, it is urgently to discover novel strategies to overcome HCC radioresistance to radiotherapies.

Several studies may provide the molecular clues of radioresistance in cancer cells. In irradiated cells, pro-survival and anti-apoptotic responses (the inducible radioresistance paradigm) would be potential targets for radiosensitization [40, 41]. Among the signaling pathways related to pro-survival and anti-apoptotic responses, NF-κB mediates the transcription of some anti-apoptosis or pro-survival regulators, *e.g.* Survivin or cIAPs in response to the activated Notch-1 pathway [40, 41]. Kang et al., 2013 and Jia et al., 2016 showed that inhibition of Notch-1/NK-κB pathway via a small molecular compound Rhamnetin leaded to a decreased radioresistance or multi-drug resistance of human cancers [23, 40]. Therefore, inhibition of NF-κB pathway activation would be a useful strategy to enhance the efficacy of radiotherapy.

The zinc finger protein A20 is a negative regulator of NF-κB signaling pathway. It can restrict NF-κB pathway's activation [42, 43]. Previous research work of A20 were mainly focused on its roles in autoimmune and inflammatory diseases. Fan et al., 2016; Catrysse et al., 2016; Nguyen et al., 2006 indicated that alteration of A20 activity may associate with the progression hepatitis C virus, hepatitis B virus and chronic liver inflammation [44–46]. In the presence work, this is the first time to reveal A20 can enhance the radiosensitivity of HCC cells to ^60^Co-γ ionizing radiation (IR). First, lower level of A20 protein was found in HCC cells compared with L-02 cells and overexpressing A20 promotes the IR-mediated injuries of HCC cells or tumor model. Second, A20 enhances IR-mediated DSB and related cell death. Third, A20 reduced the protein level of pro-survival/anti-apoptosis regulators Survivin or cIAP-1/2. A20 would also inhibit the EMT-process of HCC cells. These findings suggest that A20 may be a potential therapeutic target for HCC radiotherapy.

Currently, the reports of A20 in HCC are rare and there is not a universal role of A20 in HCC. A20 would be a potential tumor-suppressor in HCC [47]. Chen et al., 2015 suggested that A20 may suppresses HCC cells’ proliferation and metastasis through inhibition of Twist1 and exist higher expressed in HCC tissues or cell lines [47]. However, Dong et al., 2012, Wang et al., 2011 and Wang et al., 2016 showed that A20 may be a positive regulator of HCC cells’ survival or proliferation [48–50]. Targeting A20 may attenuate HCC cells’ proliferation or metastasis and protect cells from injury induced by TNFα [48–50]. In this work, our data indicated that A20 was lower expressed in HCC cells or clinical specimens, and did not significantly attenuate the proliferation or survival of HCC cells. These data may be inconsistent with each other. More works should be done in future to unmask the exact roles of A20 in HCC.

Moreover, microRNA (miRNA, miR) is a kind of 18-25 nucleotides small non-coding RNA. In mammal cells, miRs are transcribed by RNA-Polymerase III and regulate of the proliferation, metabolism and apoptosis of cells by targeting mRNA degradation [51, 52]. Expression of miRs via virus may be a strategy for gene-therapy [53–56]. Huang et al., 2016 reported that microRNA-19b-3p may modulate sensitivity of nasopharyngeal carcinoma (NC) to IR [57]. This work support our work from another point of view. It is valuable to further identify the miRs related with A20 in HCC regulation.

## MATERIALS AND METHODS

### Cell culture and adenovirus vectors

L-02, HepG2, MHCC-97H (a highly aggressive HCC cell line) and MHCC-97L (a lowly aggressive HCC cell line) cells were purchased from cell resources center of Chinese Academy of Medical Sciences (Beijing, China) and cultured in DMEM medium (Invitrogen, Carlsbad, CA, USA) added with 10% FBS at 37°C with 5% CO_2_. The adenovirus vectors containing full length sequence of A20 and its A20 siRNA was purchased from Vigene Company (Jinan, Shandong Province, China).

### HCC samples and patients

The HCC specimens and the adjacent non-tumor tissues were obtained by routinely surgical operations and preserved by our lab. The collection of tissues and the study protocol was all approved by the Ethics Committee of Xi’an Jiaotong University School of Medicine, and with the informed consent of patients. Paired HCC and non-tumorous tissues (40 paired) were obtained from surgical resections from February 2015 to April 2016. The tumor stage was identified according to Barcelona Clinic Liver Cancer (BCLC) staging classification system and histological scores were graded by Edmondson's grading system as follows: (1) well differentiated, grade I or I–II; (2) moderately differentiated, grade II or II–III; (3) poorly differentiated, grade III or III–IV. Base line characteristics were summarized in Table 1.

### qPCR analysis

Total RNA samples were extracted from clinical specimens by PARISTM Kit according to the manufacturer's instructions (Applied Biosystems, Foster City, CA, USA). RNA was reverse-transcribed into cDNA by a Multiscribe™ Reverse Transcriptase kit (Applied Biosystems, Foster City, CA, USA). Real time PCR (qPCR) was performed following the primers and protocol provided by Li et al [31].

### Western blot analysis

The antibodies against A20, Survivin, c-IAP-1, c-IAP-2 and GAPDH were purchased from Abcam cooperation. Anti-rabbit or anti-mouse IgG antibodies conjugated with horseradish peroxidase (HRP) were purchased from Sigma (St. Louis, MO, USA). Cells, which were transfected with plasmids, were then harvested for western blots. Total protein samples were extracted from HCC cells and performed by SDS-PAGE, and trans-printed to poly-vinylidene fluoride (PVDF) membranes (Millipore, Billerica, MA, USA). The membranes were blocked and then incubated with primary antibodies against A20 (1:2000 dilution), Survivin (1:500), c-IAP-1 (1:500), c-IAP-2 (1:500) or GAPDH (1:5000). The blots were then incubated with the HRP-conjugated secondary antibodies (1:5000). At last, blots were developed with enhanced chemiluminescence reagents (Pierce, Rockford, IL, USA) by X-ray films.

### ^60^Co-γ ionizing radiation and colony formation

HCC cells were treated with indicated 0, 2, 4, 6, 8 or 10Gy dose of ^60^Co-γ ionizing radiation [58, 59]. Next, 2 × 10^3^ cells per well were seeded in six-well plates (Corning, Lowell, MA, USA). After cultured for two to four weeks, the colonies of cells were identified by staining with crystal violet (0.5% diluted in 20% ethanol). Cells were harvested and measured by a multifunctional micro-plate reader at 546nm. The relative colony number = *O.D.* 546 administration group/*O.D.* 546 control group.

### TUNEL reaction

HepG2 cells treated with IR were seeded into 96-well black bottom plate (8000 cells per well) (Corning USA). 30min after IR treatment, the cells were fixed with 3% paraformaldehyde for 30 minutes and then permeablized by 0.5% Triton X-100 (diluted in PBS) for 10mins at 4°C. Then, the plates were performed by TUNEL reaction (Roche, Basel, Switzerland) as described elsewhere [60, 61]. The DSB of HepG2 cells were marked by FITC (green fluorescence signals) and the nuclear of HepG2 cells was marked by Hoechst33342 (blue fluorescence signals). All fluorescence signals were visualized by microscopy.

### Apoptosis analysis

HepG2 cells infected with control virus or A20, were treated with 6Gy dose of ^60^Co-γ IR. For apoptosis analysis, 48h after IR, cells were harvested and labeled with FITC-Annexin V and 7-AAD manufacturer's instructions (BD Biosciences, Franklin Lakes, NJ, USA). For cell cycle analysis, 48h after 6Gy dose of ^60^Co-γ IR treatment, cells were harvested and labeled with PI according to manufacturer's instructions (BD Biosciences, Franklin Lakes, NJ, USA). Cells were detected by the FACScalibur Flow Cytometer (Becton Dickinson, BD Biosciences, Franklin Lakes, NJ, USA).

### Transwell analysis

MHCC-97H cells, which were treated without or with a middle dose of ^60^Co-γ irradiation (6 Gy), were analyzed by transwell assays performed in 24-well plates chamber (Corning, Lowell, MA, USA) fitted with a polyethylene terephthalate filter membrane with 8-μm pores. The invasion-transwell or migration-transwell was performed following the methods descripted by Li et al., 2017 [62].

### *In vivo* tumor growth

All animal experiments were reviewed and approved by the Institutional Animal Care and Use Committee (IACUC) of the Xi’an Jiaotong University School of Medicine. Severe combined immune deficiency (SCID) nude mice (4–6 weeks of age) were purchased from the Animal Center, Xi’an Jiaotong University School of Medicine (Xi’an, Shaanxi, China).

For subcutaneous tumor model [63], HepG2 cells infected with control virus or A20 were treated with 6Gy dose of IR. One day after IR treatment, cells were harvested and injected into nude mice (1 × 10^6^ cells per animal). After 4-6 weeks growth, tumor volume and weight were measured. The volume of tumors was calculated: width^2^ × length / 2.

For liver *in situ* tumor model [63, 64], HepG2 cells infected with control virus or A20 were treated with 6Gy dose of IR. One day after IR treatment, HepG2 cells (1 × 10^5^ cells) were directly inoculated into the right lobe of the liver. After 4-8 weeks growth, mice were examined by a PET/CT scanner (Philips Corp., Holland). Each mouse were injected intravenously with 3.7MBq (100μCi) of ^18^F-FDG (^18^F radio-labeled fluorodeoxyglucose). Two-minute CT and Ten-minute emission scans were performed 45 minutes after injection. The radioactivity of organs and blood was measured using a NaI (Tl) well counter (China Atom Corp.).

### Statistical analysis

The western blot results were analyzed by the ALPHA INNOTECH software. Relative protein level was calculated as: (indicated group protein level / loading control level) / (control group protein level / loading control level). All statistical significance were calculated by SPSS statistical software. P-value of <0.05 was considered statistical significant.

## CONCLUSION

Our results showed A20 downregulates the protein level of cellular stress/injury response regulators and by then potentiate the susceptibility of HCC cell line to ^60^Co-γ IR. Overexpression of A20 via an adenoviral vector can be a useful strategy for increasing tumor radiosensitivity.

## SUPPLEMENTARY MATERIALS FIGURES



## Abbreviations

IR: ionizing radiation
HCC: hepatocellular carcinoma
TNFAIP3: TNFα induced protein 3
ESLD: end-stage liver diseases
cIAPs: cellular inhibitor of apoptosis
HRP: horseradish peroxidase
PVDF: poly-vinylidene fluoride
DSB: Double Strand Break
CRT: 3D-conformal RT
IGRT: image-guided RT
RFA: radiofrequency ablation
TACE: transcatheter arterial chemoembolization
EMT: epithelial-mesenchymal transition
