# Supplementary material for: A20 enhances the radiosensitivity of hepatocellular carcinoma cells to 60Co-γ ionizing radiation
Source: Oncotarget. 2017 Oct 16;8(54):93103–16. doi: 10.18632/oncotarget.21860 (PMC5696247; doi:10.18632/oncotarget.21860)
Supplement: Supplementary file 1 [file oncotarget-08-93103-s001.pdf]

A20 enhances the radiosensitivity of hepatocellular carcinoma cells to ^{60}Co-γ ionizing radiation

SUPPLEMENTARY MATERIALS

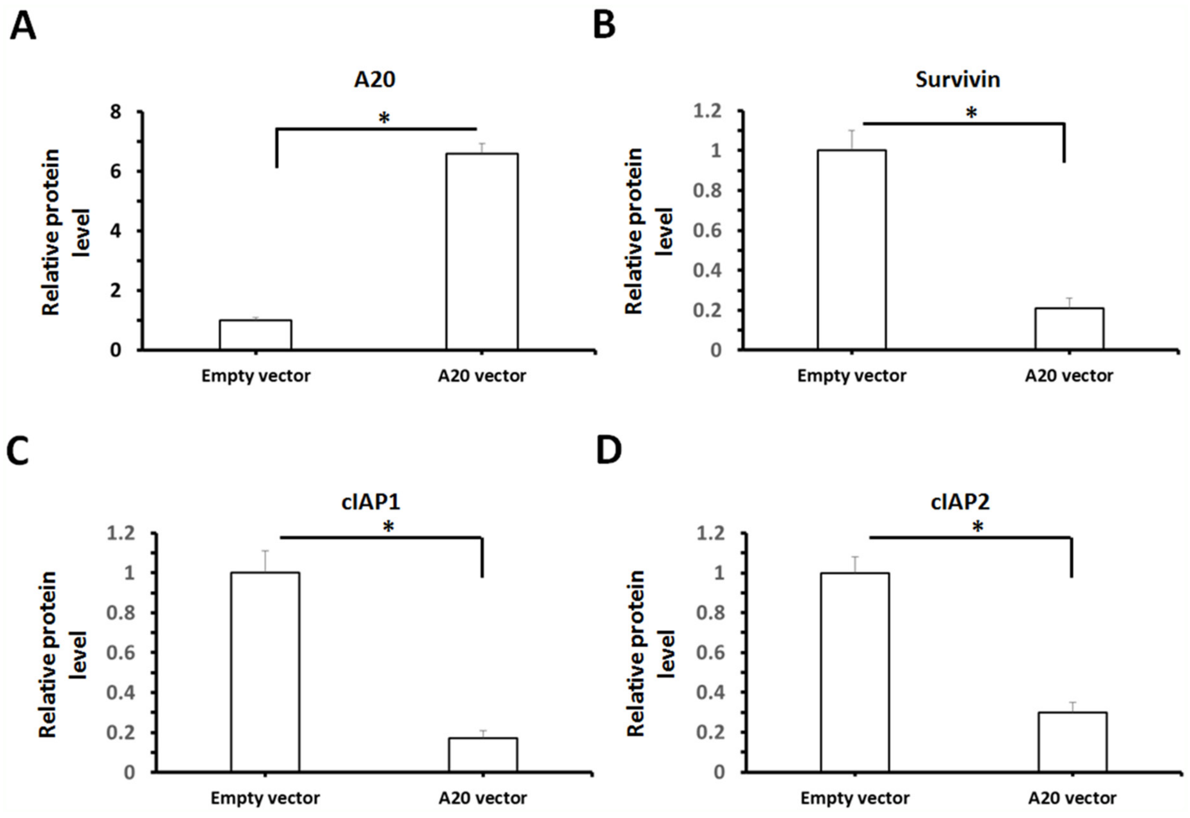

Supplementary Figure 1: The quantitative result of Figure 1 D. Quantitative results of gray scale scanning. Relative protein level of A20 **(A)**, Survivin **(B)**, cIAP-1 **(C)** or cIAP-2 **(D)** was shown. Relative protein level was shown as mean ± SD from triple experiments with similar results. $^{*}P<0.05$.

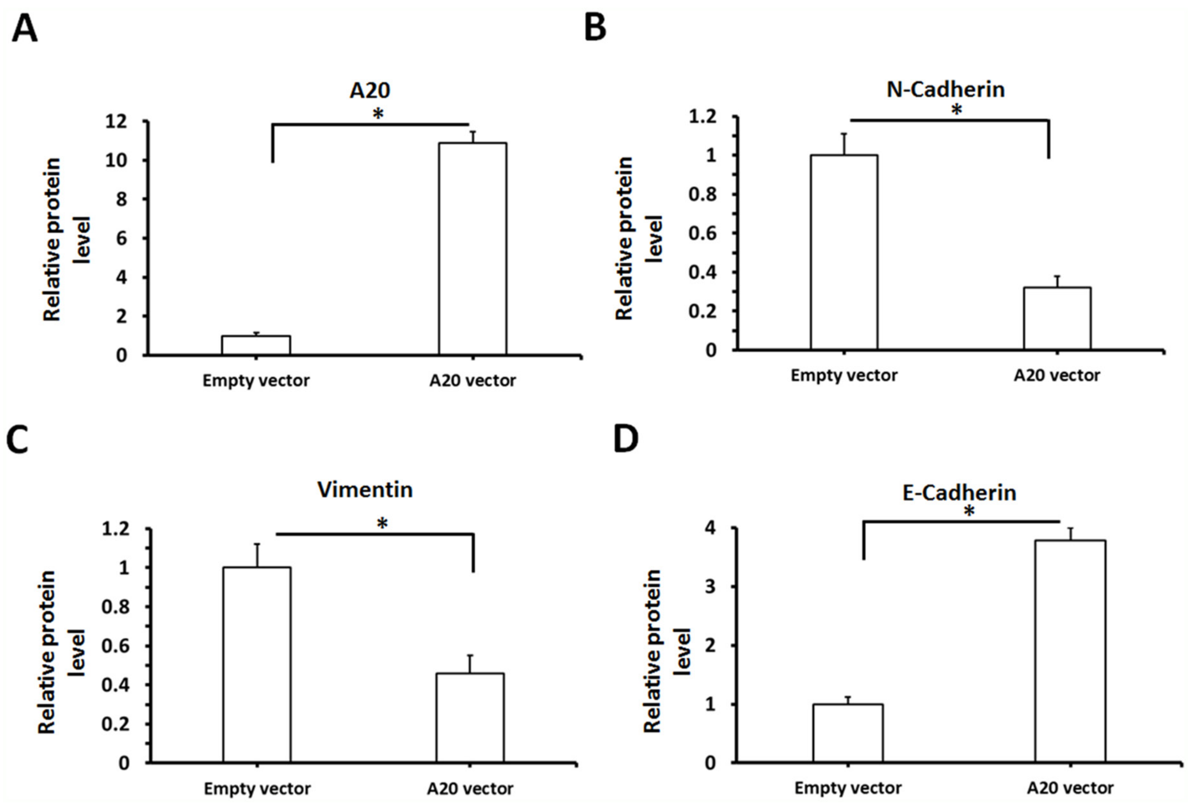

Supplementary Figure 2: The quantitative result of Figure 1 E. Quantitative results of gray scale scanning. Relative protein level of A20 **(A)**, N-cadherin **(B)**, Vimentin **(C)** or E-Cadherin **(D)** was shown. Relative protein level was shown as mean ± SD from triple experiments with similar results. $^{*}P<0.05$.

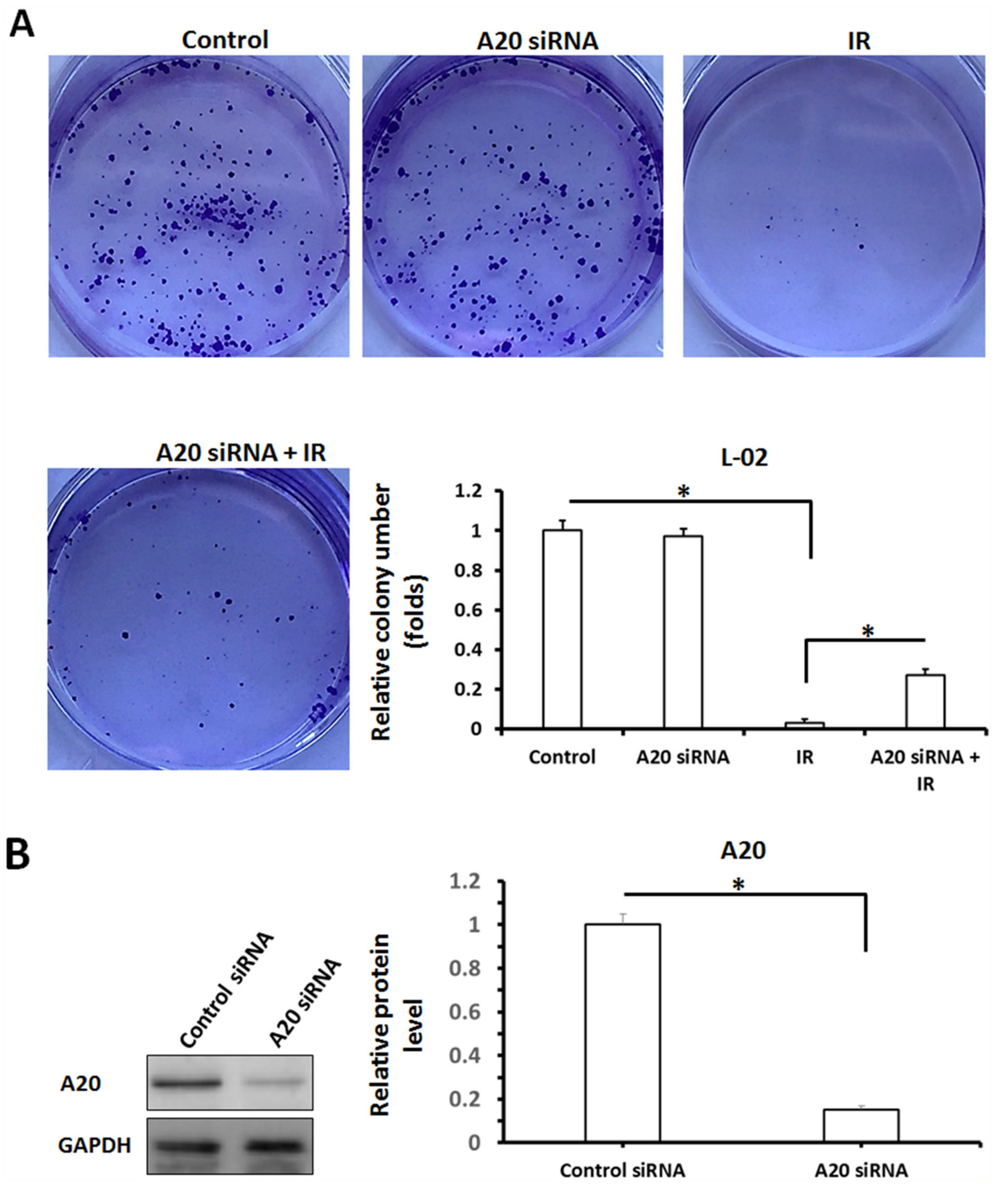

Supplementary Figure 3: Knockdown of A20 expression in L-02 cells decreased the sensitivity of HepG2 cells to ^{60}Co-γ IR. (A) HepG2 cells infected with empty vectors or A20 vectors were treated with 6Gy, a middle effect dose, of ^{60}Co-γ IR, were harvested and seed in 6-well plates (2 × 103 cells per well). After cultured for 3-4 weeks, colonies were staining with crystal violet (0.5% in 20% ethanol). Then, colonies were harvested and measured by a multifunctional micro-plate reader at 546 nm. Results were shown as (A) typical photographs or relative colony number (mean ± SD). The knockdown of A20 expression in L-02 cells were shown as western blot **(B)**. *P<0.05.

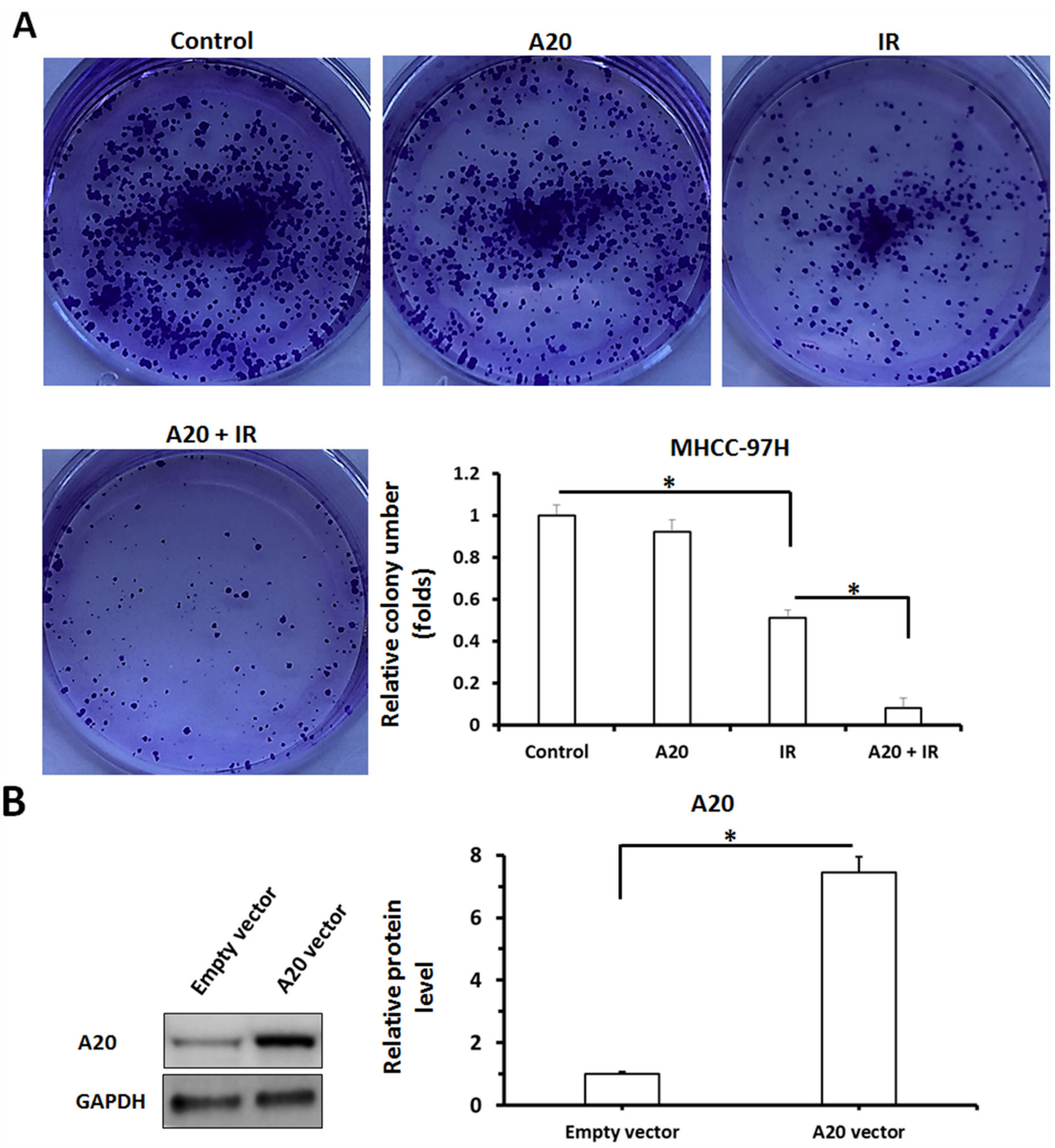

Supplementary Figure 4: Overexpression of A20 expression in MHCC-097H cells enhances the sensitivity of MHCC-97H cells to 60Co-γ IR. (A) MHCC-97H cells infected with empty vectors or A20 vectors, were treated with 6Gy dose of ^{60}Co-γ IR were harvested and seed in 6-well plates (2×10^3 cells per well). After cultured for 3–4 weeks, colonies were staining with crystal violet (0.5% in 20% ethanol). Then, colonies were harvested and measured by a multifunctional micro-plate reader at 546 nm. Results were shown as (A) typical photographs or **(B)** relative colony number (mean ± SD). Overexpression of A20 expression in MHCC-97H cells were shown as western blot (B). *P<0.05.
